# Maternal perception of children's nutritional status in the Federal District, Brazil

**DOI:** 10.1371/journal.pone.0176344

**Published:** 2017-04-26

**Authors:** Jéssica Pedroso, Natacha Toral, Muriel Bauermann Gubert

**Affiliations:** Postgraduate Program in Human Nutrition, University of Brasília, Brasília, Federal District, Brazil; Indiana University Bloomington, UNITED STATES

## Abstract

Maternal perception of child's nutritional status has a potential impact on the identification, prevention, and treatment of childhood overweight. Thus, the aim of this study was to evaluate the prevalence of misperception and factors associated with maternal perception of the nutritional status of first- to third-grade elementary school students from private schools in the Federal District, Brazil. This cross-sectional study was conducted with 554 mother-child pairs. Children's nutritional status was assessed by measuring their weight and height. The mothers completed an online questionnaire about sociodemographic data, maternal nutritional status, maternal perception of her own nutritional status (silhouette scale for female adults), and maternal perception of child's nutritional status (silhouette scale for children). Only 30.0% of the mothers were successful in choosing the most appropriate silhouette to represent child's nutritional status. Highly educated mothers (Adjusted OR = 1.51) and mothers of male children (Adjusted OR = 2.53) or of non-overweight children (Adjusted OR = 1.65) were more likely to underestimate child's nutritional status. Conversely, mothers below 35 years of age (Adjusted OR = 1.85) and mothers of female children (Adjusted OR = 2.24) or of overweight children (Adjusted OR = 1.94) were more likely to overestimate child's nutritional status. There was a high prevalence of misperception, which shows the need for interventions for children that take into account the relevance of mother's role and the adequate recognition of child's nutritional status.

## Introduction

The increased prevalence of childhood overweight and obesity is considered a public health problem in Brazil and worldwide [1, 2]. It was estimated in 2014 that 41 million children under five years old were overweight or obese, with increasing rates in middle- and low-income countries [2]. In Latin America, Rivera et al. [3] estimated that 3.8 million children under five years old and 22,2–25,9 million school-age children were overweight or obese. The last national population survey showed that 33.5% of children from 5 to 9 years of age were overweight and 14.3% were obese [4]. These findings deserve special attention, since childhood obesity is directly associated with persistence of this condition into adulthood and with a greater occurrence of associated comorbidities [5].

The most important determinants that cause childhood obesity include eating habits and sedentary lifestyle [5]. Eating habits and preferences built during childhood persist for life, and family has a strong influence on children's diet and lifestyle [6,7]. Thus, parents play a key role in preventing overweight and obesity among children [5,8].

In this context, the appropriate perception of children's nutritional status by their parents (especially their mother) becomes essential for the early recognition of childhood overweight and obesity [8–10]. Mothers usually have a greater responsibility over children's diet and education, and their perception of child's nutritional status has shown to have an influence on maternal attitudes and practices related to child's food intake [10–12].

Previous studies have found a high prevalence of inadequate maternal perception of children' nutritional status and revealed that mothers of overweight children tend to underestimate their child' nutritional status and thus be unconcerned about the consequences of childhood overweight [4, 10, 13]. Hochdorn et al. [14] verified in a systematic review that this occurs globally, and that most of the studies carried out in Latin America, East Asia and Europe noted underestimation of the nutritional status of overweight and obese children. Furthermore, many mothers believe that childhood overweight is a sign of good health and that overweight will be resolved later as the child grows [10]. With the recurrent increase in the prevalence of overweight and obesity among children and adolescents, mothers may also consider overweight as normal, especially if there are many individuals with this condition in their family or community [8].

Given the importance of maternal perception about the nutritional status of their children and its potential impact on the food offered to the child and on the identification and management of childhood overweight and obesity, the aim of the present study was to evaluate the prevalence of misperception and factors associated with maternal perception of the nutritional status of first- to third-grade elementary school students from private schools in the Federal District, Brazil.

## Material and methods

A cross-sectional study was conducted with a final sample of 554 mother-child pairs whose children were enrolled in private schools in the Federal District, Brazil. The sample is representative of first- to third-grade elementary school students from private schools in the Federal District, assuming a maximum error of 5% and 95% confidence interval and considering the sample universe as the number of children enrolled in 2013 [15].

In Brazil, usually higher social classes, attend private schools while lower socio-economic classes attend public schools. Families whose children study in private schools have a higher income and their parents have a higher level of education in comparison to those from public schools [16, 17]. In Brazil, 25.4% of the students attending elementary public schools live in households with a monthly per capita income of up to US$71, while only 3.3% of those studying in private schools lived in these conditions [16]. Therefore, private schools were chosen due to our option to use an online questionnaire. These families are more likely to access computers and Internet at home and/or work, which was necessary to fill the questionnaire. In addition, the higher educational level of the mothers helps understanding the questionnaire, allowing it to be completed independently without the aid of the researchers.

Schools selected from a previously generated random list were invited to participate in the study until reaching the minimum sample size (estimated at 474 children, considering sample power and a loss of up to 20% of questionnaires).

The Federal District, where the capital of Brazil (Brasilia) is located, is currently divided into 31 administrative regions with different characteristics, especially in relation to income and educational level of the population. This difference occurs especially when comparing the Plano Piloto and the remaining administrative regions, as citizens of the former one are usually white, middle/high income and with higher educational level, while inhabitants of the latter one are usually black, low income and have lower educational levels. Nineteen schools located in 11 different administrative regions were included in the sample, and data collection took place from April 2015 to November 2015. All students attending from first to third grades in the selected schools (and their respective mothers) were eligible and were invited to participate in the study.

Inclusion criteria for the study were children formally enrolled in the selected schools and living with their mothers. Additionally, mothers should have access to the Internet, since the questionnaire was administered online. We excluded pairs whose children had conditions that directly interfered with nutritional status, such as metabolism error, Turner syndrome, Hashimoto’s thyroiditis, diabetes mellitus, phenylketonuria, and celiac disease, or physical disabilities that limited anthropometric assessment with the equipment used in the study (scale and stadiometer) or whose mothers were pregnant. Pairs whose mothers did not fill the questionnaire completely or whose children did not have their weight and height measured were also excluded.

### Data collection

Firstly, mothers received a printed letter inviting them to participate in the study and containing the link to an online questionnaire available on the Survey Monkey platform and a code generated to identify each eligible child (and that made it possible to link the questionnaire to the anthropometric results). Before starting to complete the questionnaire, mothers were provided with the online informed consent form in which they agreed to participate in the study and consented to the participation of their child. In order to facilitate the access to the questionnaire, some schools also sent invitations to mothers by email.

Subsequently, on a day previously scheduled with the school, an anthropometric assessment was performed with children whose mothers signed the informed written consent form, agreeing with their child’s participation in the study. This situation occurred only when the child agreed to participate in the study too by signing the informed written consent form. This study was approved by the Research Ethics Committee of the School of Health Sciences at University of Brasília under the no. 39116314.3/0000.0030.

### Measurement of children's weight and height

Children's height and weight were measured using a Dayhome digital scale with maximum capacity of 150 kg and accuracy of 0.1 kg and a Stanley portable stadiometer with capacity of 2 m and graduated in centimetres. Subsequently, body mass index (BMI) was assessed. Nutritional status was classified based on BMI-for-age (BMI/age), according to the cutoff points proposed by the World Health Organization [18], using the Anthro plus software [19]. Children's anthropometric data were linked by code to the respective questionnaires answered by their mothers.

### Online questionnaire

A pilot test was performed with mothers of first- to third-grade elementary school students attending private schools not selected to investigate questionnaire's adequacy. The online questionnaire was completed by mothers and aimed to collect sociodemographic data, maternal nutritional status, maternal perception of her own nutritional status, and maternal perception of her child's nutritional status. The following sociodemographic variables were assessed: child's age and sex; maternal age, educational level, marital status, and skin colour; and family income in minimum wages (equivalent to US$209.60 at the time of the study).

Maternal nutritional status was assessed based on mother's self-reported weight and height, a procedure that has been validated and used in annual population inquiries conducted in Brazil [20–22]. These data were used to calculate maternal BMI. Maternal nutritional status was classified according to the cutoff points proposed by the WHO [23].

Maternal perception of her own nutritional status was assessed using a silhouette scale for female adults [24]. This scale was developed in Brazil and showed 15 silhouettes ranging from very thin (silhouette 1 –mean BMI = 12.5 kg/m^2^) to severely obese (silhouette 15 –mean BMI = 47.5 kg/m^2^). Firstly, maternal BMI was matched with its corresponding silhouette, which was named actual maternal silhouette (AMS). Subsequently, mothers were asked to identify, among the 15 silhouettes, the one that best represented their current body, which was named perceived maternal silhouette (PMS). Then, the agreement between AMS and PMS was assessed to investigate the presence of misperception of maternal nutritional status. Any difference between silhouettes at this stage was categorized as misperception of maternal nutritional status. When PMS was lower than AMS, mothers were considered to underestimate their own nutritional status, and when PMS was higher than AMS, they were considered to overestimate their own nutritional status.

Maternal perception of child's nutritional status was assessed using the silhouette scale for children, also developed in Brazil [24]. This scale showed 11 female silhouettes and 11 male silhouettes ranging from very thin (silhouette 1 –mean BMI = 12.0 kg/m^2^) to severely obese (silhouette 11 –mean BMI = 29.0 kg/m^2^). Firstly, the actual children's BMI was matched with its corresponding silhouette, which was named actual child's silhouette (ACS). Subsequently, mothers were asked to identify, among the 11 silhouettes, the one that best represented the current body of their child, which was named perceived child's silhouette (PCS). Then, the agreement between ACS and PCS was assessed to investigate the presence of misperception of child's nutritional status. When PCS was smaller than ACS, mothers were considered to underestimate child's nutritional status, and when PCS was bigger than ACS, they were considered to overestimate child's nutritional status. Misperception was classified as 1) mild when the difference between ACS and PCS was ±one silhouette; 2) moderate when the difference was ±two silhouettes; and 3) severe when the difference was equal to or higher than ±three silhouettes.

### Statistical methods

Pairs with underweight children were excluded from the analyses because of their low prevalence (n = 4, prevalence 0.72%). Initially, descriptive analyses were performed by calculating mean, standard deviation (SD) and frequency distribution. Data distribution was checked for normality using the Kolmogorov-Smirnov and Shapiro-Wilk tests. Since data were found to be not normally distributed, nonparametric analyses were conducted. The kappa index was used to evaluate the agreement between AMS and PMS and between ACS and PCS, considering the cutoff points proposed by Landis & Koch [25].

For bivariate and multivariate analyses, results for some variables were grouped as follows: child's age was grouped into three categories: 5–6 years, 7 years, and 8–9 years; child's and maternal nutritional status were classified as non-overweight and overweight (BMI above 25 kg/m^2^ for mothers and BMI/age above the 85th percentile for children); maternal age was classified as below or equal to 35 years old or equal to or above 36 years old; marital status was classified as: married/living with a partner or single-parent household (single/divorced/separated/widowed); maternal educational level was classified as: complete higher education and below or postgraduate education and above; maternal skin colour was classified as: white and non-white; family income was grouped into below nine minimum wages, from nine to 15 minimum wages, and above 15 minimum wages; maternal misperception of her own nutritional status was dichotomously classified as underestimated and overestimated.

The chi-square test was used for bivariate analysis to evaluate the association of sociodemographic, maternal and child's variables with outcome variables: presence/absence of maternal underestimation and overestimation of child's nutritional status.

Subsequently, a multivariate analysis with logistic regression was performed to calculate unadjusted and adjusted prevalence ratios and 95%CI. Models included variables with p ≤ 0.20 for the association with the presence/absence of underestimation and overestimation in the bivariate analysis.

The variables child's sex, child's nutritional status, maternal educational level, and maternal misperception of her own nutritional status were used as control variables in the model for the presence/absence of underestimation. In turn, the model for the presence/absence of overestimation used the control variables child's sex, child's nutritional status, maternal age, maternal educational level, and maternal misperception of her own nutritional status.

The level of significance was set at 5% and confidence interval was set at 95% (95%CI). Analyses were conducted using the Statistical Package for the Social Sciences software version 20.0.

## Results

### Descriptive analysis

Children's mean (±SD) age was 7.12 years (±0.85) and maternal mean age was 37.57 years (± 5.17). With regard to nutritional status, 21.1% of children were overweight and 12.8% were obese, according to BMI-for-age, whereas the prevalence of overweight and obesity among mothers was 28.3% and 11.2%, respectively (data not shown in tables). Most mothers participating in the study were white (64.4%), married or living with a partner (87.2%), and had a family income above nine minimum wages (68.4%) (Table 1).

**Table 1:** Descriptive analysis of sociodemographic and nutritional status data on 554 first- to third-year elementary school students of private schools and their respective mothers. Brasília (DF). 2015.

Study variables	n	%
**Child's sex**		
** Male**	280	50.5
** Female**	274	49.5
**Child's nutritional status**		
** Non-overweight**	366	66.1
** Overweight**	188	33.9
**Maternal nutritional status**		
** Non-overweight**	335	60.5
** Overweight**	219	39.5
**Maternal age**		
** ≤ 35 years**	193	34.8
** ≥ 36 years**	361	65.2
**Maternal marital status**		
** Single/divorced/separated/widowed**	71	12.8
** Married/living with a partner**	483	87.2
**Maternal educational level**		
** Complete higher education and below**	279	50.4
** Postgraduate education and above**	275	49.6
**Maternal skin colour**		
** White**	357	64.4
** Non-white**	197	35.6
**Family income** ^**a**^		
** < 9 minimum wages**	175	31.6
** 9–15 minimum wages**	142	25.6
** > 15 minimum salaries**	237	42.8

^a^: minimum wage at the time of the study: 788.00 Brazilian reais, equivalent to US$ 209.60.

### Maternal perception of child's nutritional status

It was observed that only 30.0% of mothers chose the appropriate silhouette to represent the ACS, evidencing a slight agreement between ACS and PCS (kappa = 0.150, 95%CI [0.104–0.194], p <0.001) (Table 2).

**Table 2:** Maternal perception about the nutritional status of 554 first to third-year elementary school students of private schools, according to type and level of misperception. Brasília (DF). 2015.

	Did not misperceive nutritional status	Type and level of misperception				Total
		Underestimated nutritional status^a^		Overestimated nutritional status^b^		
		Mildly	Moderately or severely	Mildly	Moderately or severely	
	n (%)	n (%)	n (%)	n (%)	n (%)	n (%)
**Overall sample**	166 (30.0)	164 (29.6)	69 (12.4)	97 (17.5)	58 (10.5)	554 (100.0)
**Child's nutritional status**						
** Normal weight**	111 (30.3)	121 (33.1)	48 (13.1)	61 (16.7)	25 (6.8)	366 (100.0)
** Overweight**	36 (30.8)	34 (29.1)	12 (10.2)	24 (20.5)	11 (9.4)	117 (100.0)
** Obesity**	19 (26.8)	9 (12.7)	9 (12.6)	12 (16.9)	22 (31.0)	71 (100.0)

^**a**^ n = 233, prevalence = 42.0%

^**b**^ n = 155, prevalence = 28.0%.

We found that 28.0% of mothers overestimated child's nutritional status, whereas 42.0% underestimated it. With regard to the level of misperception, there was a higher prevalence of mild misperception (47.1%) (Table 2).

### Maternal perception according to child's nutritional status

Our results showed that 46.2% of mothers of children classified as normal weight according to BMI/age underestimated the nutritional status of their child, 13.1% of which in a moderate or severe level, whereas 23.5% of these mothers overestimated it (Table 2).

Similar values were found for mothers of overweight children, with 39.3% underestimating the nutritional status of their child and 29.9% overestimating it. Mothers of obese children showed a higher prevalence of misperception compared with the other mothers, since 73.2% did not identify the ACS appropriately (Table 2). Of these, 25.3% underestimated the nutritional status of their child, whereas almost half overestimated it (47.9%). This overestimation was moderate or severe in 31.0% of the cases.

### Maternal perception of her own nutritional status

The prevalence of maternal misperception of her own nutritional status was high, because only 17.3% of mothers chose the appropriate silhouette to represent the AMS, evidencing a slight agreement (kappa = 0.016, 95%CI [0.033–0.096], p <0.001) (data not shown in tables). It was found that 67.9% of mothers overestimated their nutritional status and 14.8% underestimated it by choosing a PMS smaller than that corresponding to their AMS (data not shown in tables).

### Bivariate analyses of maternal perception of child's nutritional status

When cases of underestimation were compared with the remaining sample (i.e., mothers who did not misperceived child's nutritional status and those who overestimated it) (Table 3), maternal underestimation of child's nutritional status was shown to be associated with child's sex (X_1_^2^ = 23.63, p<0.001), child's nutritional status (X_1_^2^ = 7.50, p<0.01), maternal educational level (X_1_^2^ = 4.51, p = 0.03), and maternal misperception of her own nutritional status (overestimation of nutritional status) (X_1_^2^ = 4.21, p = 0.04) (Table 3).There were no associations of this variable with maternal skin colour (p>0.05) (Table 3).

**Table 3:** Biavriate association of sociodemographic and maternal variables with the presence/absence of underestimation and overestimation of child's nutritional status. Brasília (DF). 2015.

Variables	Underestimated	Did not underestimate	*p*	Overestimated	Did not overestimate	*p*
	n (%)	n (%)		n (%)	n (%)	
**Child's sex**			<0.001			<0.001
**Male**	146 (52.1)	134 (47.9)		58 (20.7)	222 (79.3)	
**Female**	87 (31.8)	187 (68.2)		97 (35.4)	177 (64.6)	
**Child's age**			0.41			0.63
**5–6 years**	55 (37.4)	92 (62.6)		45 (30.6)	102 (69.4)	
**7 years**	89 (43.8)	114 (56.2)		57 (28.1)	146 (71.9)	
**8–9 years**	89 (43.6)	115 (56.4)		53 (26.0)	151 (74.0)	
**Child's nutritional status**			<0.01			<0.01
**Non-overweight**	169 (46.2)	197 (53.8)		86 (23.5)	280 (76.5)	
**Overweight**	64 (34.0)	124 (66.0)		69 (36.7)	119 (63.3)	
**Maternal nutritional status**			0.30			0.66
**Non-overweight**	135 (40.3)	200 (59.7)		96 (28.7)	239 (71.3)	
**Overweight**	98 (44.7)	121 (55.3)		59 (26.9)	160 (73.1)	
**Maternal age**			0.58			< 0.01
**≤ 35 years**	78 (40.4)	115 (59.6)		70 (36.3)	123 (63.7)	
**≥ 36 years**	155 (42.9)	206 (57.1)		85 (23.5)	276 (76.5)	
**Marital status**			0.58			0.60
**Single/divorced/separated/widowed**	32 (45.1)	39 (54.9)		18 (25.4)	53 (74.6)	
**Married/living with a partner**	201 (41.6)	282 (58.4)		137 (28.4)	346 (71.6)	
**Maternal educational level**			0.03			0.06
**Complete higher education and below**	105 (37.6)	174 (62.4)		88 (31.5)	191 (68.5)	
**Postgraduate education and above**	128 (46.5)	147 (53.5)		67 (24.4)	208 (75.6)	
**Maternal skin colour**			0.46			0.57
**White**	146 (40.9)	211 (59.1)		97 (27.2)	260 (72.8)	
**Non-white**	87 (44.2)	110 (55.8)		58 (29.4)	139 (70.6)	
**Family income**			0.92			0.89
**< 9 minimum wages**	72 (41.1)	103 (58.9)		48 (27.4)	127 (72.6)	
**9–15 minimum wages**	59 (41.5)	83 (58.5)		42 (29.6)	100 (70.4)	
**> 15 minimum wages**	102 (43.0)	135 (57.0)		65 (27.4)	172 (72.6)	
**Maternal misperception of her own nutritional status**			0.18			0.03
**Underestimated**	40 (48.8)	42 (51.2)		15 (18.3)	67 (81.7)	
**Did not underestimate**	193 (40.9)	279 (59.1)		140 (29.7)	332 (70.3)	
**Maternal misperception of her own nutritional status**			0.04			0.17
**Overestimated**	147 (39.1)	229 (60.9)		112 (29.8)	264 (70.2)	
**Did not overestimate**	86 (48.3)	92 (51.7)		43 (24.2)	135 (75.8)	

When cases of overestimation were compared with the remaining sample (i.e., mothers who did not misperceive child's nutritional status and those who underestimated it), maternal overestimation of child's nutritional status was shown to be associated with child's sex (X_1_^2^ = 14.82, p<0.001), child's nutritional status (X_1_^2^ = 10.75, p<0.01), maternal age (X_1_^2^ = 10.10, p<0.01), and maternal misperception of her own nutritional status (underestimation of nutritional status) (X_1_^2^ = 4.48, p = 0.03) (Table 3). There were no associations of this variable with maternal skin colour (p>0.05).

### Multivariate analyses of maternal perception of child's nutritional status

Child's sex, child's nutritional status, and maternal educational level were the variables that remained significantly associated with underestimation even after adjusting the model (Table 4). It was found that the chance for male children to have their nutritional status underestimated was 153.0% higher than that of female children. Moreover, non-overweight children had a 1.65-fold higher chance of having their nutritional status underestimated. It was also observed that highly educated mothers were more likely to underestimate the nutritional status of their child (adjusted OR = 1.51) (Table 4).

**Table 4:** Unadjusted and adjusted odds ratio for underestimation and overestimation of child's nutritional status according to associated factors. Brasília (DF). 2015.

	Presence of underestimation		Presence of overestimation	
Associated factors	OR (95%CI)	Adjusted OR (95%CI)^a^	OR (95%CI)	Adjusted OR (95%CI)^b^
**Child's sex**				
**Male**	2.34	2.53	1	1
	(1.66–3.31)	(1.77–3.61)		
**Female**	1	1	2.10	2.24
			(1.43–3.07)	(1.51–3.32)
**Child's nutritional status**				
**Non-overweight**	1.66	1.65	1	1
	(1.15–2.39)	(1.12–2.41)		
**Overweight**	1	1	1.89	1.94
			(1.29–2.77)	(1.30–2.91)
**Maternal age^c^**				
**≤ 35 years**			1.85	1.85
			(1.26–2.71)	(1.23–2.77)
**≥ 36 years**			1	1
**Maternal educational level**				
**Higher education and below**	1	1	1.43	1.31
			(0.98–2.08)	(0.85–1.95)
**Post Graduate education and above**	1.44	1.51	1	1
	(1.03–2.03)	(1.06–2.16)		
**Maternal misperception of her own nutritional status**				
**Underestimated**	1.38	0.95	0.53	0.61
	(0.86–2.20)	(0.51–1.75)	(0.29–0.96)	(0.29–1.28)
**Did not underestimate**	1	1	1	1
**Maternal misperception of her own nutritional status**				
**Overestimated**	0.69	0.67	1.33	1.09
	(0.48–0.98)	(0.42–1.07)	(0.89–2.00)	(0.65–1.83)
**Did not overestimate**	1	1	1	1

OR: Odds ratio. CI: Confidence interval. Adjusted OR^a^: odds ratio adjusted by logistic regression for child's sex, child's nutritional status, maternal educational level, and maternal misperception of her own nutritional status. Adjusted OR^b^: odds ratio adjusted by logistic regression for child's sex, child's nutritional status, maternal age, maternal educational level, and maternal misperception of her own nutritional status. ^c:^ The variable maternal age was not tested for the presence of underestimation because it had p > 0.20 in bivariate analyses.

With regard to maternal overestimation of child's nutritional status, the variables sex, child's nutritional status, and maternal age remained significantly associated after adjusting the model (Table 4). It was shown that the chance for female children to have their nutritional status overestimated was 124% higher than that of male children. Moreover, overweight children had a 94.0% higher chance of having their nutritional status overestimated. It was also observed that younger mothers, i.e. below 35 years of age, had a 1.85-fold higher chance of overestimating the nutritional status of their child (Table 4).

The associations between the presence of over or underestimation and maternal misperception of her own nutritional status did not remain significant after adjustments in the logistic regression model (Table 4).

## Discussion

The prevalence of overweight children and women found in the present study are similar to those observed in previous studies, showing a high prevalence of overweight and obesity among children and women in Brazil [4,26,27].

Only one-third of the mothers chose the appropriate silhouette for their child. Previous studies show that mothers find it difficult to identify the nutritional status of their children appropriately [14, 28–30]. Molina et al. [28] found a low correspondence between maternal perception and child's nutritional status, especially for overweight children. The reasons to justify this difficulty in identifying child's nutritional status have not been completely elucidated yet, but it has been suggested that inadequate mother's perception may result from their deep concern with child's nutritional status, leading mothers to believe that their child is either under or overweight. The present study was not able to confirm this assumption, which would be a worthwhile area for further work.

A high percentage of mothers underestimated the nutritional status of their overweight children (39,3%), a trend that has been previously observed among parents of this population [8,7,12,13,14,31,32]. However, contrary to previous findings, 29.9% of mothers of overweight children and 47.9% of those of obese children overestimated the nutritional status of their child. Such a high frequency has not been found in previous studies, which may be explained by the method used to evaluate maternal perception in our study (silhouette scale). Lazzeri et al. [33] revealed that the use of silhouette scales tends to reduce the percentage of mothers who underestimate the nutritional status of obese children compared with the use of multiple-choice questions, in which mothers were asked to classify the nutritional status of their child as underweight, normal weight, overweight or obesity. These questions may make mothers reluctant to identify their children as overweight, because of emotional factors, and thus lead them to underestimate child's weight. Hence, when using the silhouette scale, mothers feel more comfortable to choose the child's silhouette without needing to classify the child into categories [13,29,33,34].

It was also found that 46.2% of mothers of normal weight children underestimated child's nutritional status, whereas 23.5% of these mothers overestimated it. Both underestimation and overestimation of the nutritional status of normal weight children are concerning because they may lead to maternal dissatisfaction with the supposed child's thinness or overweight and affect the way mothers behave regarding dietary intake in order to promote weight gain or loss in health children [10,11,32].

Maternal perception influences children's feeding control practices, a relationship that may be mediated by maternal concerns about child's nutritional status [35]. Mothers who perceive their child as either underweight or overweight tend to show greater concern. Previous studies found that the greater mother's concern about child's overweight, the most inclined she is to restrict child's diet. Conversely, mothers who are concerned about child's underweight tend to press the child to eat [35]. Thus, maternal perception of the nutritional status of their children impacts the quantity and quality of foods offered to children [28]. Restriction and pressure-to-eat practices can negatively impact individual's dietary intake and are associated with a lower control over innate hunger and satiety cues [36]. Although the present study found a high prevalence of maternal misperception of child's nutritional status, it did not explore the association of this misperception with child's dietary intake and controlling food-related parenting practices, an issue that may be investigated in further studies.

In this context, evaluating the low correspondence between maternal perception and the child's nutritional status and its possible consequences, Hochdorn et al. [14] highlighted the role of education in prevention. Therefore, it is relevant to emphasize the importance of governmental initiatives that seek to prevent overweight in childhood and to help families to recognize the nutritional status of their children properly. Currently, in Brazil the public health system guarantees access to consultation with pediatricians and nutritionists, seeking the prevention and treatment of overweight in childhood, based on evaluation of nutritional status and the promotion of healthy eating. Also there are programs that promote health inside public schools such as the Health Program at School (promotes, among other activities, healthy eating and assessment of the children’s nutritional status) and the National School Feeding Program (promotes food and nutrition education activities and the provision of adequate meals during the period in which children remain in school) [37,38] In addition, the intersectoral strategy for the prevention and control of obesity seeks to prevent and control obesity in the Brazilian population, through intersectoral actions, promoting adequate and healthy food consumption and the practice of physical activity in the environment we live [39]. However, these initiatives should also seek the accurate recognition of the nutritional status of children by their families and include the participation of private schools, which are usually not included in their strategies.

Furthermore, a high percentage of mothers was found to overestimate their own nutritional status. The way women perceive their own body image may influence the perception about the nutritional status of their children and maternal attitudes towards this status [10]. However, the present study did not find an association between maternal perception of her own nutritional status and her perception of child's nutritional status after adjusting the models.

With regard to child's sex, it could be seen that boys were more likely to have their nutritional status underestimated by their mothers, whereas girls were more likely to have it overestimated. Other studies also found an association between child's sex and maternal perception of nutritional status [12,28,32,34]. Mothers tend to be more concerned about the nutritional status of their daughters, wishing them to be thinner. This may reflect the beauty standard imposed by the media and may lead to severe consequences, such as an increased prevalence of eating disorders [10,32,40]. Conversely, with regard to boys, it has been suggested that the greater trend of mothers to underestimate child’s nutritional status may be explained by the idealization of strong and robust bodies for male individuals, but additional studies are needed to evaluate these aspects [41].

Non-overweight children are more likely to have their nutritional status underestimated, whereas overweight children are more likely to have it overestimated. Aparício et al. (2013) [10] found in a previous study that child's BMI was a predictor of maternal perception, since the greater child's BMI, the larger the silhouette chosen by the mother.

It was found that highly educated mothers were more likely to underestimate the nutritional status of their child, which differs from results usually observed in other studies, in which less educated mothers were more likely to misperceive the nutritional status of their children [12,28,42,43]. This difference may be justified by the high educational level of all mothers participating in this study, since private education is expensive in Brazil, and only middle and high income families are able to keep their children at private schools. Other studies evaluated populations with more heterogeneous educational levels, which does not allow for a strict comparison of studies.

It was observed that younger mothers were more likely to overestimate the nutritional status of their children compared with mothers older than 35 years of age. Giacomossi et al. (2011) [43] showed that mothers from 24 to 35 years of age had a lower prevalence of error in the classification of child's nutritional status compared with mothers younger than 24 years of age. Conversely, Aparício et al. (2013) [10] observed that mothers belonging to an older age group (≥ 40 years) were more likely to underestimate child's nutritional status.

There were no associations of maternal overestimation and underestimation of child’s nutritional status with maternal skin color. In our study only 35.6% of the mothers were non-white, while in Brazil and in the Federal District, 52,3% and 57,8% of the population declare themselves to be non-white, respectively [44]. Thus, our study does not represent the racial distribution observed in the Federal District and Brazil as a whole [44]. Future studies should be encouraged to assess the racial grouping using an adequate sample.

The limitations of this study are the mother’s self reported weight and height, as well as the cross-sectional design, which does not allow the establishment of causal relationships. Also, the selection of the sample from private schools limits the extrapolation and the broader applicability of the results due to the huge population of Brazil, and great socioeconomic differences between private and public schoolers and their families.

## Conclusion

The present study found a high prevalence of maternal misperception of child's nutritional status. Moreover, highly educated mothers, mothers of boys, and mothers of non-overweight children were more likely to underestimate child's nutritional status. On the other hand, younger mothers, mothers of girls, and mothers of overweight children were more likely to overestimate child's nutritional status.

Thus, in view of the complexity of the topic and the low correspondence between maternal perception and child's nutritional status, there is a need for interventions that take into account the relevance of mother's role and that help her recognize the nutritional status of her children appropriately. It is also worth highlighting that the implementation of strategies to prevent or treat childhood overweight should be focused not only on children but also on their mothers, providing guidance to help promote an accurate view of a healthy weight for their children.

## Supporting information

S1 Dataset Maternal perception of child’s nutritional status dataset. (XLSX)
